# Eight to Sixteen

**Published:** 1878-09

**Authors:** 


### EIGHT TO SIXTEEN.

Lord Shaftesbury recently stated in a public meeting in London, that from personal observation he had ascertained, that of the adult male criminals of that city, nearly all had fallen into a course of crime between the ages of eight and sixteen years; and that if a young man lived an honest life up to twenty years of age, there were forty-nine chances in his favor, and only one against him, as to an honorable life thereafter.

Thus is it in the physical world. Half of all who are born, die under twenty years of age, while four fifths of all who reach that age, and die before another "score," owe their death to causes of disease which were originated in their "teens." On a careful inquiry, it will be ascertained that in nearly all cases, the causes of moral and premature physical death, are pretty much one and the same, and are laid between the ages of "eight and sixteen years." This is a fact of startling import to fathers and mothers, and shows a fearful responsibility. Certainly a parent should secure and retain and exercise absolute control over the child until sixteen; it can not be a difficult matter to do this, except in very rare cases, and if that control is not wisely and efficiently exercised, it must be the parent's fault; it is owing to parental neglect or remissness. Hence the real source of ninety eight per cent of the crime of a country such as England or the United States, lies at the door of the parents. It is a fearful reflection; we throw it before the minds of the fathers and mothers of our land, and there leave it, to be thought of in wisdom, remarking only as to the early seeds of bodily disease, that they are nearly in every case sown between sundown and bed-time, in absence from the family circle, in the supply of spending-money never earned by the spender, opening the doors of confectioneries and soda-fountains, of beer and tobacco and wine, of the circus, the negro minstrel, the restaurant and the dance; then follow the Sunday excursion, the Sunday drive, with easy transition to the company of those whose ways lead down to the gates of social, physical and moral ruin. From "eight to sixteen!" in these few years are the destinies of children fixed! in forty-nine cases out of fifty; fixed by the parent! Let every father and every mother, solemnly vow: "By God's help, I'll fix my darling's destiny for good by making home more attractive than the street."

